# Micro-Ultrasound Imaging for Accuracy of Diagnosis in Clinically Significant Prostate Cancer: A Meta-Analysis

**DOI:** 10.3389/fonc.2019.01368

**Published:** 2019-12-10

**Authors:** Minhao Zhang, Rong Wang, Yuqing Wu, Jibo Jing, Shuqiu Chen, Guangyuan Zhang, Bin Xu, Chunhui Liu, Ming Chen

**Affiliations:** ^1^Surgical Research Center, Institute of Urology, Medical School of Southeast University, Nanjing, China; ^2^Department of Urology, Wuxi XiShan People's Hospital, Wuxi, China; ^3^Department of Urology, Affiliated Jintan Hospital of Jiangsu University, Changzhou, China; ^4^Department of Urology, Affiliated Zhongda Hospital of Southeast University, Nanjing, China

**Keywords:** micro-ultrasound, clinically significant prostate cancer, diagnostic accuracy, biopsy, ExactVu, meta-analysis

## Abstract

**Background:** Prostate cancer is a frequently diagnosed malignant solid tumor in men. The accuracy of diagnosis is becoming increasingly important. This meta-analysis evaluated the accuracy of micro-ultrasound in the diagnosis of clinically significant prostate cancer.

**Methods:** We searched PubMed, Embase, Web of Science, and Cochrane Library databases to recruit studies in English. The quality assessment of diagnostic accuracy studies-2 protocol was used to evaluate the literature quality. Publication bias was analyzed using Deeks' funnel plot asymmetry test. We calculated the pooled sensitivity, specificity, positive likelihood ratio (PLR), negative likelihood ratio (NLR), diagnostic odds ratio (DOR), and 95% confidence interval (95% CI) for studies of micro-ultrasound imaging for prostate cancer. The results were assessed by the summary receiver-operating characteristic curve (SROC). Ultimately, a univariable meta-regression and subgroup analysis, Fagan plot, and a likelihood matrix were conducted.

**Results:** A total of seven studies containing 769 patients were included in this meta-analysis. Micro-ultrasound had a pooled sensitivity, specificity, DOR, and an area under the SROC of 0.91, 0.49, 10, and 0.82, respectively. Based on these findings, micro-ultrasound has superior ability to diagnose clinically significant prostate cancer.

**Conclusion:** Micro-ultrasound is a more convenient and cost-effective method in real-time imaging during the biopsy procedure in detecting clinically significant prostate cancer. Although micro-ultrasound has shown promising results, more clinical data and comprehensive analysis are still needed.

## Introduction

Prostate cancer (PCa) is a frequently diagnosed malignant solid tumor in men. It is the second leading cause of cancer deaths in the United States. In 2019, 174,650 new PCa were diagnosed, and 31,620 deaths were attributed to this disease in the United States (1). These estimated new cases and deaths are significantly higher than in 2018. In recent years, PCa has become the third most common type of cancer in China, and the morbidity and mortality of PCa have steadily increased (2). The prostate-specific antigen (PSA) test and digital rectal examination (DRE) are recommended for PCa screening. Magnetic resonance imaging (MRI) techniques are also used for evaluating PCa. These techniques provide unique information that is helpful to differentiate PCa from non-cancerous tissue and have been proven to improve diagnostic accuracy (3). Conventional ultrasound-based rectal systematic biopsy is insufficient, even with repeated biopsy every 6–24 months, pathological findings suggest significant differences (4, 5). Clinically significant PCa (csPCa) was considered for any Gleason sum ≥7 and International Society of Urological Pathology (ISUP) grade >2. Rouviere et al. reported that there was no difference between systematic biopsy and MRI-targeted biopsy in the detection of PCa with ISUP grade 2 or above, but the combination of the two methods could further improve the detection rate. Multi-parameter MRI examination before biopsy can improve the detection rate of csPCa, but systematic biopsy cannot be avoided. It is controversial whether multi-parameter MRI can detect more csPCa and avoid systematic biopsies (6).

The EXACTVU™ (Exact Imaging, Markham, Canada) micro-ultrasound is a novel high-resolution 29-MHz ultrasound that offers real-time biopsies targeted to suspicious areas and enables the detailed visualization of related prostate tissue characteristics, with three times greater resolution as compared with conventional ultrasound resolution (7). The ExactVu system is located in the urologist's usual procedure room. In addition to the procedure, the targeting and the entire workflow are controlled by the urologist. Micro-ultrasound also has PRI-MUS (prostate risk identification using micro-ultrasound), a protocol for users to easily learn and quickly apply to help guide targeted biopsies to suspicious regions (8). The micro-ultrasound system is a total urological solution; it operates using conventional transducers as well as high-resolution 29-MHz biopsy transducers. Targeting is performed on the same instrument, and no MRI/fusion system is required.

Previous study indicated that micro-ultrasound may help with screening protocols by ensuring that all men with PCa are offered biopsy in a timely manner, while reducing the number of men without csPCa who are required to undergo the standard biopsy procedure (9). Therefore, we combined all those published evidences in a systematic manner to analyze the accuracy of diagnosis by micro-ultrasound for prostate biopsy.

## Materials and Methods

### Search Strategy

This meta-analysis is based on the Preferred Reporting Items for Systematic Reviews and Meta-Analyses (PRISMA) guidelines (10). The literature research was conducted via PubMed, Web of Science, Embase, and Cochrane Library databases before July 30, 2019. Keywords were “micro-ultrasound” and “prostate.” Additional records were identified through the website https://www.exactimaging.com/papers-and-publications. Two authors independently searched the databases.

### Criteria for Inclusion and Exclusion

According to the standard for reporting diagnostic accuracy studies (STARD) (11), the criteria for including studies were as follows: (1) Features of lesions cannot be determined before diagnosis; (2) The group of masses was diagnosed by micro-ultrasound; (3) The reference standard should be histopathologic diagnoses, such as biopsy or surgical pathological examination; (4) The data of four-panel (the true-positive, false-positive, false-negative, and true-negative patients) can be obtained directly or indirectly; (5) Number of patients ≥15. Exclusion criteria: (1) The reference standard is inconsistent; (2) Study design and statistical methods are improper. The published articles and the abstracts were all included in this study. The titles and abstracts of articles were independently assessed by two reviewers. The enrolled articles were evaluated and further screened by viewing the whole text.

### Data Extraction and Quality Assessment

Two researchers independently collected the required data from available studies, including the name of the first author, the year of publication, distribution of population, size of sample, csPCa patients, study type, mean age, mean PSA, mean prostate volume, true-positive, false-positive, false-negative, true-negative, sensitivity, specificity, positive prediction value, and negative prediction value, if applicable.

### Quality Assessment and Statistical Analysis

We used the quality assessment of diagnostic accuracy studies-2 (QUADAS-2) to evaluate the quality of the literature (12). According to the bivariate mixed model (13), pooled sensitivity, specificity, positive likelihood ratio, negative likelihood ratio, and DOR were conducted to determine the accuracy of micro-ultrasound for diagnosing PCa. We produced a forest plot and a summary receiver-operating characteristic curve (SROC) from all of the studies. The area under the curve (AUC) was used to describe the overall accuracy as a summary of the SROC. Non-threshold heterogeneity was evaluated by the *Q* test and *I*-squared, and *I*^2^ > 50% and *P* < 0.1 suggested an obvious heterogeneity in terms of statistics. Meta-regression and subgroup analysis were used to identify the source of heterogeneity. Fagan plot analysis was used to assess the relationship among the pretest probability of the disease, the likelihood ratio of the diagnostic test, and posttest probability of the disease. We also generated a likelihood matrix, which is represented as a scatter plot of the positive and negative likelihood ratios. We used STATA software version 15.0 (Stata Corporation, College Station, TX, USA) and Review Manager software (RevMan, Version 5.3) to analyze the data. A *P*-value < 0.05 suggested statistical significance.

## Results

### Study Characteristics

**Figure 1:**
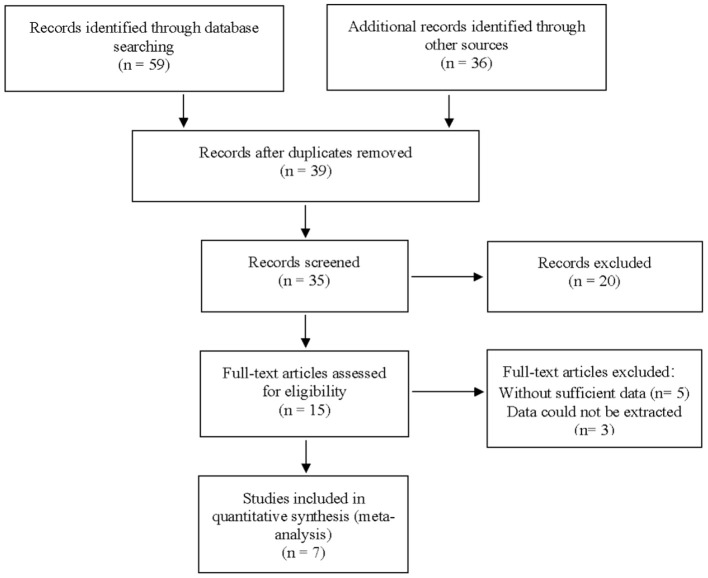
The flowchart for the identification of eligible studies.

**Figure 2:**
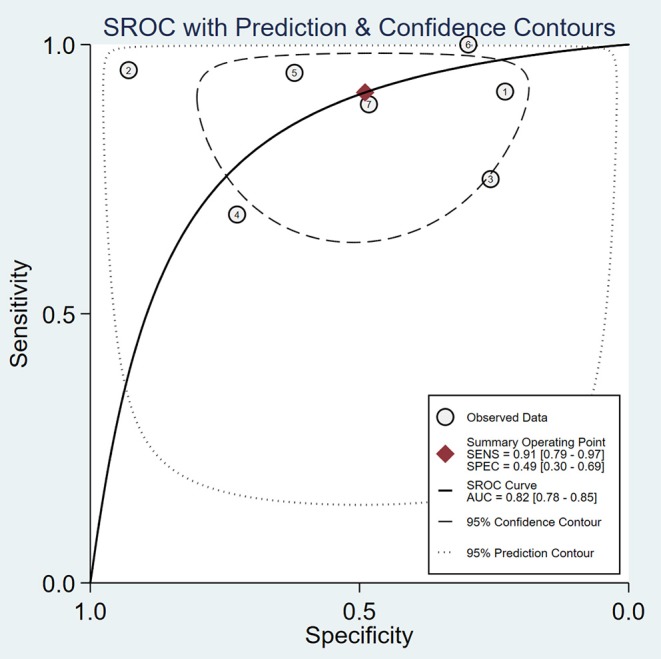
Summary receiver-operating characteristic curve (SROC) curve of micro-ultrasound area under the curve (AUC).

A total of 59 articles were identified in our literature search. Thirty-nine articles were included in this systematic review after eliminating duplicate articles. According to the inclusion criteria in the study selection process, seven articles were selected for the meta-analysis (Figure 1). The baseline characteristics of the included studies are presented in Table 1. As shown in Table 2, the data related to micro-ultrasound diagnosis of csPCa are presented. The pooled sensitivity, pooled specificity, likelihood ratios, and AUROC are provided in Table 3 and Figure 2.

**Table 1:** Baseline characteristics of included articles.

**References**	**Cases**	**Study design**	**Blinded**	**Consecutive**	**Mean age**	**Mean PSA (ng/ml)**	**Mean prostate volume (ml)**	**Reference standard**
Lughezzani et al. (14)	286	Prospective	Yes	Yes	64	9	57.3	Histology
Astobieta et al. (15)	35	Prospective	Yes	Yes	NR	NR	NR	Histology
Abouassaly et al. (16)	67	Prospective	No	Yes	66	5.37	38	Histology
Chessa et al. (17)	68	Prospective	Yes	Yes	NR	NR	NR	Histology
Claros et al. (18)	48	Retrospective	No	No	66.9	9.1	54.4	Histology
Luger et al. (19)	142	Prospective	Yes	Yes	66	5.39	NR	Histology
Eure et al. (20)	123	Retrospective	Yes	Yes	NR	NR	NR	Histology

*PSA, prostate specific antigen; NR, not reported*.

**Table 2:** Summary of results of micro-ultrasound in included studies.

**References**	**Micro-ultrasound**			
	**TP**	**FP**	**FN**	**TN**
Lughezzani et al. (14)	94	141	9	42
Astobieta et al. (15)	20	1	1	13
Abouassaly et al. (16)	21	29	7	10
Chessa et al. (17)	39	3	18	8
Claros et al. (18)	18	11	1	18
Luger et al. (19)	48	66	0	28
Eure et al. (20)	8	59	1	55

*TP, true-positive; FP, false-positive; FN, false-negative; TN, true-negative*.

**Table 3:** Pooled sensitivity, pooled specificity, pooled likelihood rations, and AUROC of micro-ultrasound.

Pooled sensitivity	0.91
(95% CI)	(0.79–0.97)
Pooled specificity	0.49
(95% CI)	(0.30–0.69)
Pooled positive LR	1.80
(95% CI)	(1.20–2.70)
Pooled negative LR	0.18
(95% CI)	(0.07–0.50)
Pooled DOR	10.00
(95% CI)	(3.00–35.00)
AUROC	0.82
(95% CI)	(0.78–0.85)

*AUROC, area under the summary receiver-operating characteristic curve; CI, confidence interval*.

### Quality Assessment in Included Studies

**Figure 3:**
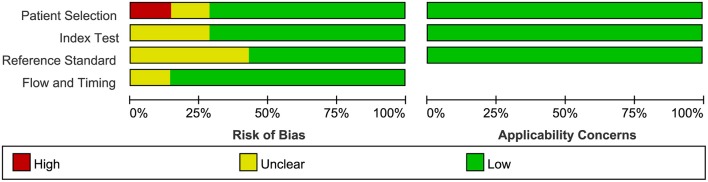
Group bar charts show risk of bias and applicability concerns of the seven included records using quality assessment of diagnostic accuracy studies-2 (QUADAS-2).

The quality of all seven available studies in our meta-analysis was evaluated based on the QUADAS-2 protocol, and the risk of bias and applicability concerns of seven studies is shown in Figure 3. In general, the quality of the included studies was considered high. Regarding the patient selection domain, Gregg's study was considered to have a high risk of bias as the included patients were not identified by pathology. Regarding the index test domain, the studies by Abouassaly et al. and Claros et al. were considered to have unclear risk because blinding was unclear. Regarding the reference standard domain, three studies had unclear risk of bias as it is uncertain whether the interpretation of the reference standard used the blind method. Regarding the flow and timing domain, the study by Abouassaly et al. was considered to have unclear risk. There was low concern for applicability with regard to the first three QUADAS-2 domains for all seven included studies.

### Heterogeneity Test and Subgroup Analysis

**Figure 4:**
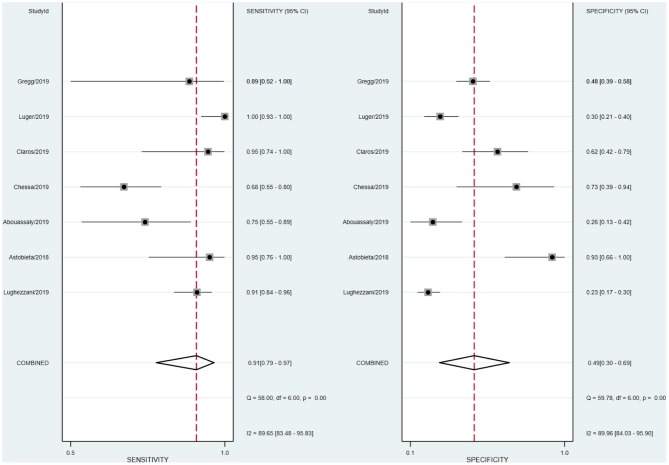
Forest plots of sensitivity and specificity of micro-ultrasound.

**Figure 5:**
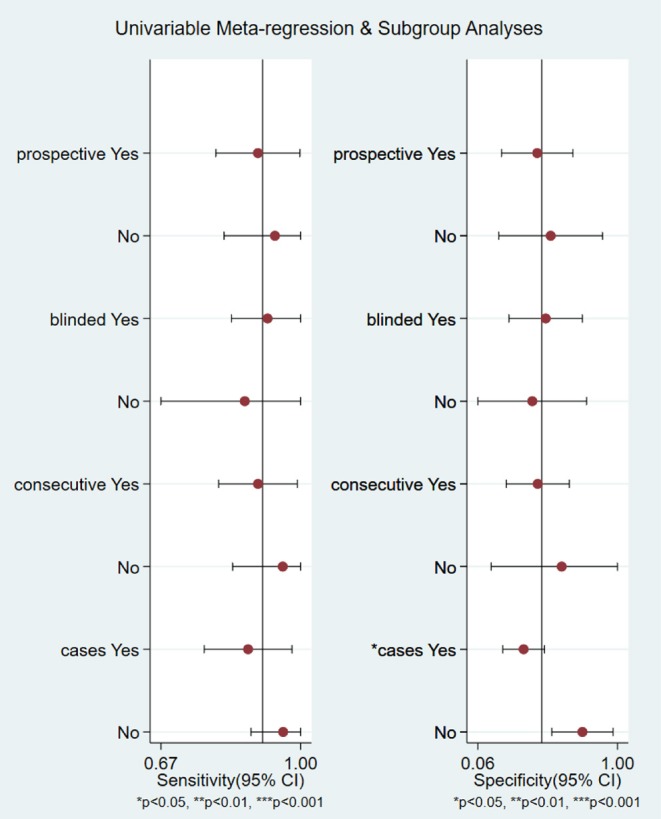
The detailed data for the univariable meta-regression analysis and subgroup analysis.

We analyzed the sensitivity and specificity of micro-ultrasound in seven studies. A *P*-value < 0.05 indicated that significant heterogeneity exists among these seven studies. As shown in Figure 4, the forest plots of micro-ultrasound indicated that the heterogeneity existed among the included articles. In addition, we used meta-regression analysis to evaluate various covariates from these studies, including the “whether blinding was applied,” “study type,” “consecutive or random,” and “cases.” The detailed data for the meta-regression analysis are presented in Figure 5. From the specificity results of micro-ultrasound, the covariates of “cases” were statistically significant. Thus, the results of this meta-regression analysis suggested that the sample size may be the source of potential heterogeneity.

### Publication Bias

**Figure 6:**
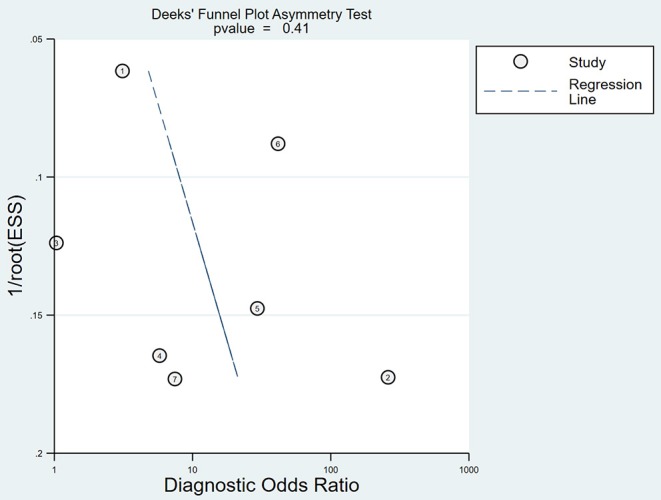
Deeks' funnel plot asymmetry test to evaluate publication bias.

The publication bias for micro-ultrasound was determined through Deeks' funnel plot. The shape of the funnel plots was almost symmetrical, suggesting low publication bias (*P* = 0.41; Figure 6).

### Fagan Plot Analysis and Likelihood Matrix

**Figure 7:**
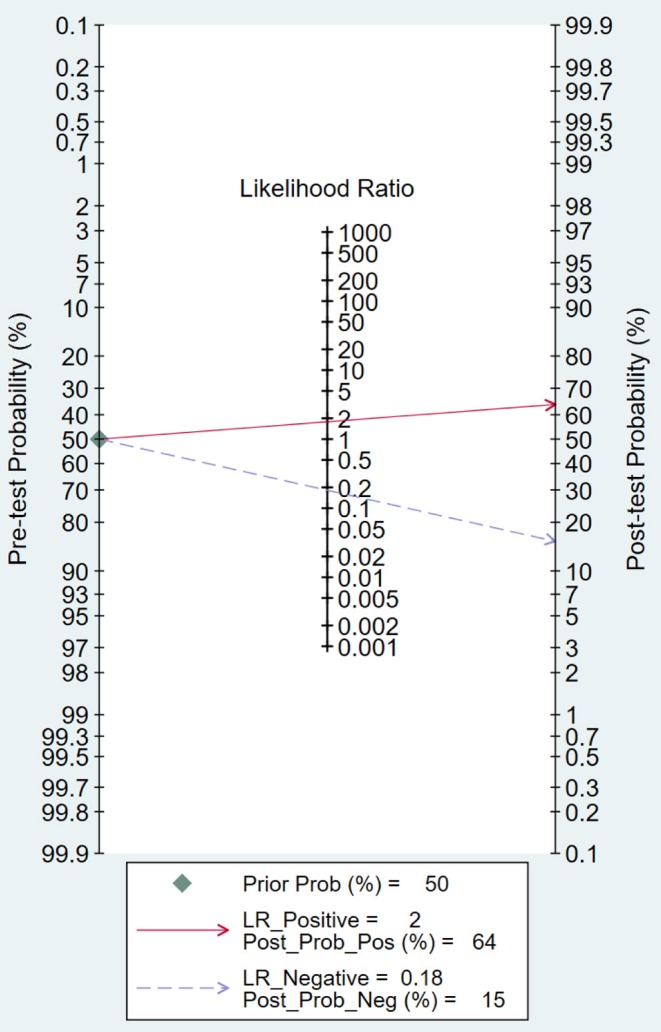
Fagan plots of micro-ultrasound by patient analysis for the diagnosis of prostate cancer.

Likelihood ratio and posttest probability are closely related to clinical disease. In our study, both the likelihood ratio and posttest probability were moderate (Figure 7). Given a pretest probability of 50%, the positive posttest probability is 64%, and the negative posttest probability is 15%.

**Figure 8:**
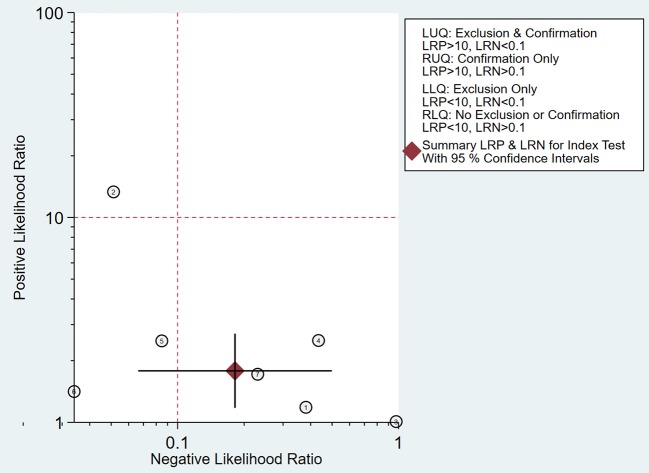
Likelihood matrix indicates that summary positive likelihood ratio (PLR) and negative likelihood ratio (NLR) for micro-ultrasound diagnosis of clinically significant prostate cancer (csPCa) with 95% confidence intervals are concentrated on the right lower quadrant (RLQ).

As shown in Figure 8, the summary PLR and NLR for micro-ultrasound diagnosis of csPCa were concentrated in the right lower quadrant. This information indicates that the PLR was <10 and the NLR was >0.1.

## Discussion

People with suspected PCa usually need prostate biopsy first, which can result in morbidity, such as bleeding, infection, and rectal and bladder injury. Increasing the positive rate of suspected prostate lesions can significantly reduce unnecessary biopsies and complications. Previous studies demonstrated that MRI has high sensitivity and specificity (21, 22). The application of MRI for prostate biopsy is a usual method, but it is not recommended as an alternative for systemic biopsy at present (23). Micro-ultrasound, as a novel high-resolution imaging method for prostate biopsy, has received increasing attention. In our meta-analysis, we evaluated the diagnostic accuracy of micro-ultrasound for csPCa. After our comprehensive and systematic literature retrieval and verification, a total of seven studies met the inclusion criteria. Micro-ultrasound's high sensitivity makes it an attractive option for guiding targeted biopsy (pooled sensitivity 91%, pooled specificity 49%). We calculated the DOR, a single indicator of test accuracy, and the mean DOR was 10, which demonstrates a high level of overall accuracy. In addition, we calculated the AUC of micro-ultrasound (AUROC 0.82), which indicated a high level of overall diagnostic accuracy.

The forest plot indicated that heterogeneity existed in sensitivity and specificity among the studies (*I*^2^ > 50%). The univariable meta-regression and subgroup analysis revealed that the covariates “cases” might be the potential source of heterogeneity with regard to specificity. In studies of micro-ultrasound by Chessa et al. and Abouassaly et al., the sensitivity was 0.68 and 0.75, respectively (16, 17). There were 57 and 28 patients in the two studies, respectively, with positive lesions among the 39 and 21, respectively, who had successful detection, leading to the lower sensitivity. In the studies written by Luger et al., Abouassaly et al., and Lughezzani et al., the specificities were 0.30, 0.26, and 0.23, respectively (14, 16, 19). Astobieta et al. (15) indicated that there were 14 patients with negative lesions among those who had successful detection, and 13 of them showed negative prediction, leading to the highest specificity of 0.93. The studies of micro-ultrasound by Claros et al. and Eure et al. showed that the specificities were 0.62 and 0.48, respectively (18, 20). The results of our meta-analysis indicated that micro-ultrasound had high sensitivity for detection of csPCa, but the specificity was moderate.

On the other hand, Fagan plot analysis is also important to determine the effectiveness of a diagnostic test. Fagan plot analysis indicated that micro-ultrasound has limited value in improving the diagnosis and exclusion of csPCa, and the result suggested that the use of micro-ultrasound cannot confirm or exclude malignancy.

A likelihood ratio plot was drawn to visually demonstrate that micro-ultrasound is effective in improving the accuracy of csPCa diagnosis. Epidemiological study (24) suggested that a pooled positive likelihood ratio >10 and pooled negative likelihood ratio <0.1 indicated diagnostic value for csPCa. However, all of the scatter points were clustered in the lower right quadrant. These results suggested that the use of micro-ultrasound cannot confirm or exclude malignancy. We need to combine additional clinical data and tests for a more comprehensive analysis.

Our meta-analysis was based on data extracted from published literature. In addition, we objectively analyzed the application of micro-ultrasound in the diagnosis of csPCa by calculating the pooled sensitivity and specificity. Fagan plot and likelihood matrix were applied to evaluate the accuracy of csPCa diagnosis. Finally, we used stratified analysis to examine the results of variables in subgroups of patients. This study has several limitations. First, there may be linguistic bias in the retrieval of English literature only. Second, there is a lack of quality assessment criteria for studies. However, to address this, our study is based on strict inclusion criteria and quality evaluation criteria; the included articles are high quality after quality assessment, and the data are reliable. No meta-analysis has ever been published in this area; the analysis methods are scientific and rigorous, which we used. Third, according to these results, micro-ultrasound has high sensitivity but poor specificity. The detection ability of micro-ultrasound is strong, but the possibility of misdiagnosis is high, and additional studies are needed to obtain better application values. The low specificity of micro-ultrasound may be due to the learning curve. However, compared with single study, the results have greater efficiency and credibility. We need large-scale studies to validate the clinical application of micro-ultrasound as a diagnostic tool for csPCa.

## Conclusion

In our meta-analysis of seven studies, micro-ultrasound is a more convenient and cost-effective method in real-time imaging during the biopsy procedure in detecting clinically significant prostate cancer. Although micro-ultrasound has promising results, more clinical data and comprehensive analysis are needed.

## Data Availability Statement

All datasets generated for this study are included in the article/supplementary material.

## Author Contributions

MZ, BX, and CL contributed to the conception and design of the study. RW organized the databases. YW and JJ performed the statistical analysis. MZ and YW wrote the first draft of the manuscript. SC, GZ, and MC wrote sections of the manuscript. All authors contributed to manuscript revision, read, and approved the submitted version.

### Conflict of Interest

The authors declare that the research was conducted in the absence of any commercial or financial relationships that could be construed as a potential conflict of interest.

## Abbreviations

PCa: prostate cancer
csPCa: clinically significant prostate cancer
mpMRI: multiparametric magnetic resonance imaging
PRI-MUS: prostate risk identification using micro-ultrasound
PLR: positive likelihood ratio
NLR: negative likelihood ratio
DOR: diagnostic odds ratio
CI: confidence interval.
